# Supplementary material for: LAGSwin: Local attention guided Swin-transformer for thermal infrared sports object detection
Source: PLoS One. 2024 Apr 9;19(4):e0297068. doi: 10.1371/journal.pone.0297068 (PMC11003622; doi:10.1371/journal.pone.0297068)
Supplement: Attachment — Submitted filename: Answer.docx [file pone.0297068.s002.docx]

PONE-D-23-13636R1

LAGSwin: Local attention guided Swin-transformer for thermal infrared sports object detection

PLOS ONE

Dear Dr. Mao,

Thank you for submitting your manuscript to PLOS ONE. After careful consideration, we feel that it has merit but does not fully meet PLOS ONE’s publication criteria as it currently stands. Therefore, we invite you to submit a revised version of the manuscript that addresses the points raised during the review process.

Please submit your revised manuscript by Jan 06 2024 11:59PM. If you will need more time than this to complete your revisions, please reply to this message or contact the journal office at [plosone@plos.org](mailto:plosone@plos.org). When you're ready to submit your revision, log on to https://www.editorialmanager.com/pone/ and select the 'Submissions Needing Revision' folder to locate your manuscript file.

Please include the following items when submitting your revised manuscript:

- A rebuttal letter that responds to each point raised by the academic editor and reviewer(s). You should upload this letter as a separate file labeled 'Response to Reviewers'.
- A marked-up copy of your manuscript that highlights changes made to the original version. You should upload this as a separate file labeled 'Revised Manuscript with Track Changes'.
- An unmarked version of your revised paper without tracked changes. You should upload this as a separate file labeled 'Manuscript'.

If you would like to make changes to your financial disclosure, please include your updated statement in your cover letter. Guidelines for resubmitting your figure files are available below the reviewer comments at the end of this letter.

If applicable, we recommend that you deposit your laboratory protocols in protocols.io to enhance the reproducibility of your results. Protocols.io assigns your protocol its own identifier (DOI) so that it can be cited independently in the future. For instructions see: <https://journals.plos.org/plosone/s/submission-guidelines#loc-laboratory-protocols>. Additionally, PLOS ONE offers an option for publishing peer-reviewed Lab Protocol articles, which describe protocols hosted on protocols.io. Read more information on sharing protocols at <https://plos.org/protocols?utm_medium=editorial-email&utm_source=authorletters&utm_campaign=protocols>.
We look forward to receiving your revised manuscript.

Kind regards,

Nattapol Aunsri, Ph.D.
Academic Editor
PLOS ONE

**Reviewer #3:**

**1.** In the discussion section, there is a reference to Figure 2 in the discussion of the results. However, Figure 2 was not located inside the document itself (revision1 version). "line 472 ---> framework, Figure 2 and Table 4 show the detection performance of the model for each". Please examine the document for clarity and completeness by ensuring that all related figures are included. This visual assistance would greatly improve understanding of your experimental outcomes as well as the efficiency of the LAGSwin method across various sports targets.

**Answer.** We are very grateful to the experts for their constructive comments. We have made detailed revisions in response to these comments.

For instance,

(2) In the TTsports dataset, the detection accuracy of each class is very competitive. It is worth noting that although $sportsman6$ and $sportsman3$ have the same detection performance, AP is $0.838$, but R is $0.919$ and $0.925$, respectively. The possible reason is that $sportsman6$ has occlusion during the movement, which reduces the R-value. A visual presentation of the different data is shown in Figure 2.

**2.** Please use proper formatting throughout the manuscript, particularly with items such as double quotation marks (" "). Check the consistency and accuracy of these symbols.

**Answer.** We are very grateful to the experts for their constructive comments. We have made detailed revisions in response to these comments.

For instance,

(1) In the RGBsports data set, compared to the baseball class whose ID number is 0, the detection effect of football is significantly better than that of baseball, namely, AP and R have increased by $0.184$ and $0.116$, respectively. The possible reason is that the shape and size of the football are easy. Distinguishing from the image background enables the network to learn a more practical difference between the target and the background, thereby improving the detection performance of football. The smaller size of the baseball is easy to confuse with the target background and causes misclassification. In the FLIRs data, the $otherscars$ class with ID number 3 achieved the worst performance, probably because this class has a small number of samples, and the slight difference between its $cars$ caused misclassification. This reduces the detection accuracy and the performance of the detection framework for the $cars$ class. For example, the APs of $dog$ and $people$ are $0.053$ and $0.069$ higher than $cars$, respectively.
